# Behavioural, Neural and Molecular Effects Underlying Caffeine's Addictive Properties

**DOI:** 10.1111/adb.70114

**Published:** 2025-12-21

**Authors:** Xiaonan Li, Fei Fei, Xiaomin Liu, Peicai Cui, Huaquan Sheng, Ying He, Yi Shen, Yihan Gao

**Affiliations:** ^1^ Shanghai New Tobacco Product Research Institute Co., Ltd. Shanghai China

**Keywords:** 3D behavioural analyses, caffeine, conditioned place preference, medial prefrontal cortex, transcriptome analysis

## Abstract

Caffeine is one of the most widely consumed psychoactive substances globally. It acts as a non‐selective adenosine receptor antagonist. Despite its extensive use, the effects of caffeine on the central nervous system remain poorly understood. This study aims to investigate the behavioural changes and underlying neural mechanisms associated with caffeine addiction. The results of conditioned place preference (CPP) revealed that caffeine induces a strong preference shift, demonstrating its rewarding properties. 3D Behavioural Profiling and Motion Analysis further demonstrated significant differences in locomotion, rearing and sniffing behaviours between caffeine‐treated and saline‐treated groups. C‐fos and fibre photometry experiments revealed that caffeine activates the medial prefrontal cortex (mPFC) region. Transcriptomic profiling revealed a significant enrichment of pathways associated with transcriptional regulation and calcium signalling in the medial prefrontal cortex (mPFC) of caffeine‐treated mice. Together, these findings provide a multidimensional perspective on caffeine's addictive properties, as well as its modulation of mPFC activity and molecular pathways. This contributes to a deeper understanding of caffeine's effects on the central nervous system.

## Graphical abstract

Caffeine‐addicted mice exhibit measurable changes in 3D behavioral patterns. The medial prefrontal cortex (mPFC) of caffeine‐addicted mice showed significant activation. Caffeine addiction induces significant alterations in gene expression in the mPFC.

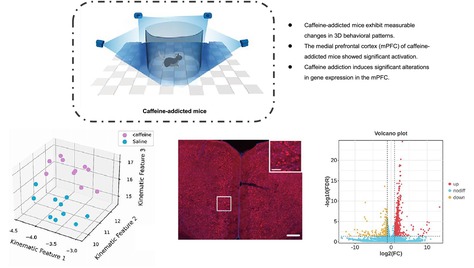

## 1 Introduction

Caffeine, a natural alkaloid found in coffee, tea and other plant‐based products, is the most widely consumed psychoactive substance worldwide [1]. Its physiological and psychological effects, including enhanced alertness [2], improved cognitive performance [3] and increased physical stamina [4], have made it an integral part of daily life for many. Despite its broad use, caffeine's psychoactive effects, particularly its potential for addiction and its mechanisms of action in the central nervous system (CNS), remain topics of considerable scientific interest [5].

Caffeine's primary mode of action involves antagonism of adenosine A1 and A2A receptors [6, 7], which are widely expressed in the brain. Adenosine, an endogenous neuromodulator, plays a critical role in promoting sleep and regulating neuronal activity [8, 9, 10]. Caffeine increases dopaminergic [11, 12] and cholinergic neurotransmission [13] in key brain regions such as the prefrontal cortex (PFC) [14] and striatum [15] through adenosine receptor antagonism, indirectly enhancing excitatory drive. These effects are thought to underlie its stimulant and reinforcing properties.

Although caffeine is generally regarded as a mild stimulant with low addiction potential compared to substances such as nicotine or opioids, emerging evidence suggests that it can induce physiological and psychological dependence with continual higher doses of caffeine [16]. Behavioural studies, such as conditioned place preference (CPP) [17] and operant conditioning, have been instrumental in evaluating the reinforcing properties of caffeine. Additionally, advances in neuroimaging [18], electrophysiology [19] and transcriptomics [20] offer powerful tools for exploring the neural and molecular mechanisms underlying caffeine's effects.

This study aims to provide a comprehensive evaluation of caffeine's impact on behaviour, neural circuits and molecular pathways associated with addiction. Using a combination of behavioural assays, neural activation recording and transcriptomic analyses, we investigate the multidimensional effects of caffeine on the mPFC. Based on these findings, subsequent comparisons with the addictive properties of other substances can be conducted, thereby providing a theoretical foundation for investigating both shared and distinct mechanisms among different addictive drugs.

## 2 Materials and Methods

### 2.1 Experimental Animals

Adult male mice were C57BL/6 mice (8–10 weeks) weighing 22–28 g (Shanghai Jihui Experimental Animal Breeding Co. Ltd.), which were housed for at least 1 week in advance in an animal room with alternating cycles of 12 h of light and 12 h of darkness (lights on at 7:00 am and off at 7:00 pm), at a temperature of 22°C–26°C and 40%–60% humidity. All mice had free access to food and water. All experimental procedures were approved by the Animal Ethics and Use Committee of Fudan University (approval number: 20241107‐001) and were performed in accordance with NIH guidelines. Mice were randomly divided into cages and labelled according to experimental groups.

### 2.2 CPP

The CPP model was utilised to assess the rewarding properties of the alkaloids [21]. The experimental procedure began with a preconditioning phase where animals were acclimated to the laboratory environment and allowed to explore the CPP apparatus freely for 15–20 min to reduce novelty‐induced stress and identify their natural compartment preferences. This phase served to establish a baseline for comparison during subsequent testing. In the conditioning phase, animals received intraperitoneal injections of alkaloid solutions or saline according to their group assignment. During morning sessions, to determine the effective working concentration, we dissolved caffeine at three different concentrations (0.16, 0.32, 0.64 mg/kg) in saline and administered it via intraperitoneal injection at a volume of 0.2 mL per 20 g of mouse body weight for CPP modelling. Alkaloid‐treated animals were confined to their non‐preferred compartments for 30 min to associate the environment with drug‐induced euphoria. After 12 h, saline injections were paired with the preferred compartments to attenuate natural bias. This process was repeated for 5 days. Twenty‐four hours later, in the postconditioning phase, animals were allowed to explore the apparatus freely without any treatment for 15 min. Time spent in each compartment was recorded, and the CPP score, defined as the difference in time between compartments, was calculated. The software recorded the time spent by the mice in each chamber respectively, and the corresponding CPP scores were subsequently calculated manually. A higher CPP score indicated the preference for the caffeine‐paired context, demonstrating the rewarding effects of caffeine.

### 2.3 The 3D‐Motion Capture System

The 3D‐motion capture system was used to capture the movements of mice during the spontaneous behavioural test [22]. The open‐field box is made up of a transparent acrylic wall that stands 30 cm tall and a white plastic square floor with sides measuring 40 cm in length. Although a small cuboid (15 cm in length, 18 cm in width and 15 cm in height) was present in one corner of the open field box, an acrylic transparent partition was placed at the junction of the two boxes to prevent the mouse from accessing the small cuboid freely. The open field arena was positioned at the center of a movable stainless‐steel support framework measuring 130 × 130 × 90 cm^3^. The framework had four Intel RealSense D435 cameras mounted orthogonally on its four supporting pillars, and a 56‐in. screen was placed horizontally but face down on the top of the shelf to provide uniform and stable white background light. Animals' behavioural data were extracted from 16 key body parts, including the nose, left ear, right ear, neck, left front limb, right front limb, left hind limb, right hind limb, left front claw, right front claw, left hind claw, right hind claw, back, root tail, middle tail and tip tail. These key points were used to reconstruct 3D skeletons. We measured the speed and movement energy of different body parts in mice as comparative parameters for analysis, while also comparing the limb‐constituted movements as kinematic parameters. The detailed methods, including camera calibration and animals' behavioural image acquisition, of this 3D multi‐view motion‐capture system setup (BA‐DC01, Shenzhen Bayone BioTech Co. LTD, Shenzhen), were described in our previous work. Movement fractions mean that when mice are active in the recording chamber, they often exhibit repeated, stereotyped behaviours. During the analysis, these specific actions are identified and extracted, with their timing and frequency being quantified.

### 2.4 Immunostaining

Ninety minutes after the final CPP test, animals were perfused with 4% paraformaldehyde, and their brains were sectioned into 40‐μm slices. Brain slices were blocked and incubated with primary antibodies targeting the c‐Fos protein [23], followed by secondary and tertiary antibody incubations. Stained slices were visualised using fluorescence microscopy, and c‐Fos‐positive cells in key brain regions were quantified to evaluate neuronal activation across groups.

### 2.5 Stereotaxic Surgery

The surgical procedure was as follows: the mice were anaesthetised with isoflurane (2 L V%V); the surgery was performed under a continuous gas mixture of oxygen and isoflurane. The mouse's head was adjusted to a horizontal position by Bregma and Lambda points on a stereotaxic instrument. Small holes were drilled above the mPFC brain region (AP: 1.8, ML: 0.5, DV: 1.5), and bleeding was promptly stopped if it occurred. The virus was injected as a dopamine neurotransmitter probe: rAAV_2/9_‐hSyn‐GCaMP6f‐WPRE‐pA, with an injection volume of 300 nL and an injection rate of 30–50 nL/min, and the needle was stopped for 10 min after the injection to allow complete virus diffusion. The optical fibre was buried above the mPFC brain region of the mice with a specific optical fibre holder, and the optical fibre was fixed with dental cement. After the dental cement was completely dried, the mice were removed from the operating table and when the mice were awakened, they were marked and put back into the feeding cage.

### 2.6 Fibre Photometry Recording

After 3 weeks of virus expression, the fibre optic patch cord of the fibre optic recording system was fixedly connected to the ceramic insert to test the fluorescent signal, and animals that responded well were prepared for the test. GCaMP6f signals were observed in mice before and after the injection of caffeine. The value of ΔF/F = (F‐F0)/F0 was used to characterize the change in fluorescence intensity around the event as a response to the change in neuron activity under caffeine. The recorded data were exported as mat files for further analysis in Matlab software, first preprocessed by steps such as (1) baseline calibration, (2) down sample and (3) smooth. The signal change was calculated following the following formula: ΔF/F = (F‐F0)/(F0‐Foffset). The significance of the parameters in this formula is as follows: F represents the current signal value; F0 represents the mean signal value 15 min before drug administration; Foffset represents the background noise value of the instrument; ΔF/F represents the relative signal change value, expressed as a percentage; and the percentage signal change before and after stimulation was plotted for a single mouse using the Matlab software plot function.

### 2.7 Transcriptomic Analysis

After the CPP tests, brain tissues from mPFC regions were rapidly dissected and flash‐frozen. RNA was extracted and processed to construct cDNA libraries, which were sequenced using high‐throughput technology. Differentially expressed genes were identified, and pathway enrichment analyses were performed to map the molecular pathways and networks involved in alkaloid‐induced addiction [24].

### 2.8 Analysis

Numerical data were expressed as mean ± SEM. Off‐line data analysis was performed using software GraphPad Prism 6 (GraphPad Software, USA). The normality of the data has been tested. When the assumption was not satisfied, non‐parametric tests were conducted. Otherwise, comparisons were conducted with Student's *t* test or two‐way ANOVA followed by Bonferroni's post‐tests for multiple comparisons where appropriate. *n* refers to the number of mice. Every group of mice in each experiment was from at least 5 animals. For all results, *p* < 0.05 was considered statistically significant. *p < 0.05, **p < 0.01, ***p < 0.001 and ****p < 0.0001.

## 3 Results

### 3.1 Caffeine‐Induced CPP

**FIGURE 1:**
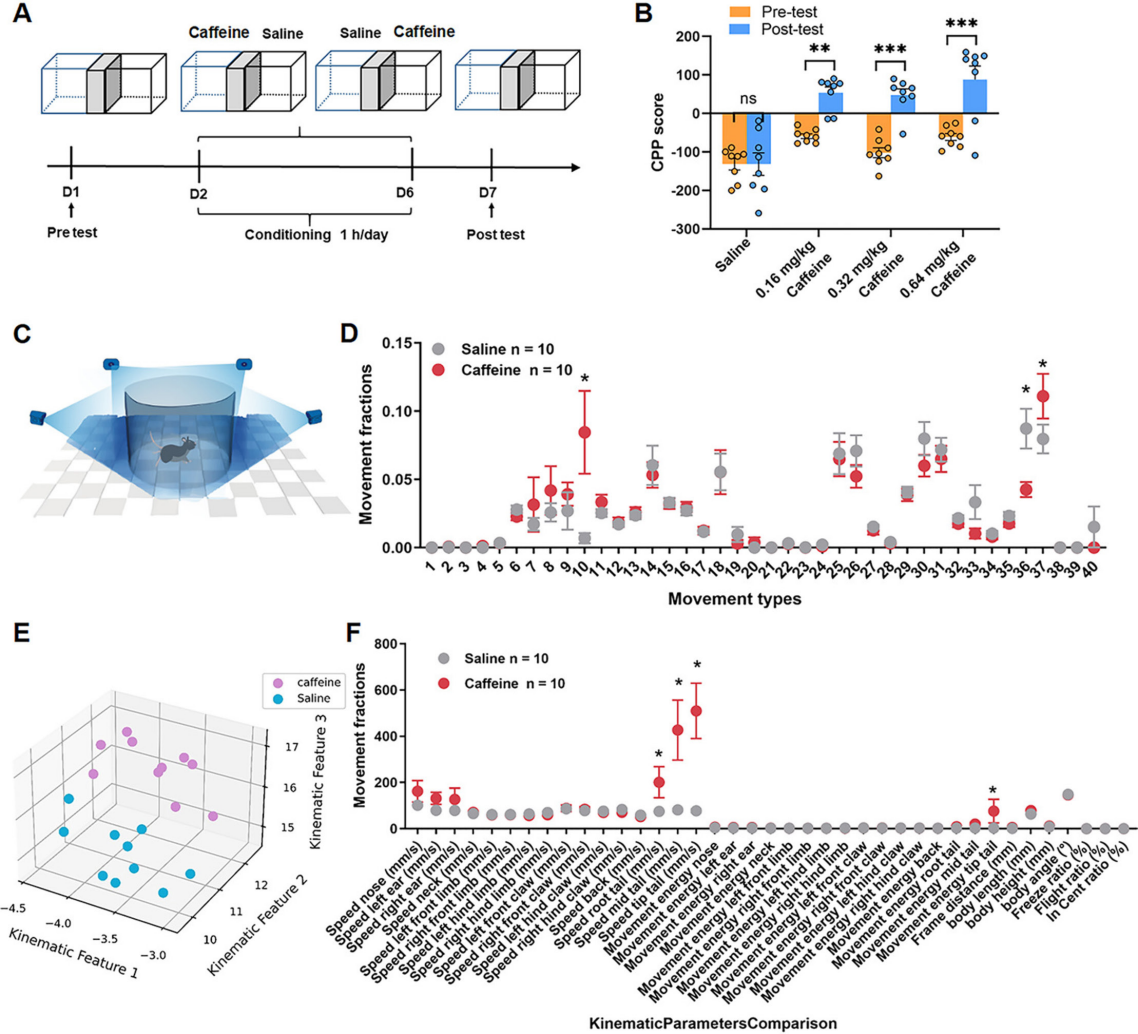
Behavioural effects of caffeine in adult male mice. (A) Schematic of the experimental setup. (B) The average CPP score in three dose of caffeine groups (Two‐way ANOVA, *p* < 0.05, Sidak's multiple comparisons test, the low dose, *p* < 0.05, the medium dose, *p* < 0.01, the high dose, *p* < 0.01, *n* = 8 in 0.16 mg/kg concentration group, *n* = 10 in 0.32 mg/kg concentration group, *n* = 8 in 0.64 mg/kg concentration group). (C) Schematic diagram of recording animal behaviour with four synchronised cameras. (D) Movement fractions of 40 movement types in 3D behavioural analysis in the saline group and caffeine group. (E) Low‐dimensional representation of the two animal groups (saline group, *n* = 10; caffeine group, *n* = 10). The 20 dots in 3D space were dimensionally reduced from 40‐dimensional movement fractions, and they are well separated. (F) Movement fractions of kinematic parameters comparison in 3D behavioural analysis in the saline group and caffeine group. *p < 0.05, **p < 0.01, ***p < 0.001, Means ± SEMs.

To determine the optimal caffeine concentration for CPP modelling (Figure 1A), mice were conditioned with low (0.16 mg/kg), medium (0.32 mg/kg) and high (0.64 mg/kg) doses of caffeine. Behavioural responses were assessed before (pre‐test) and after (post‐test) conditioning. The result of CPP is shown in the Table 1. There are significant differences between pre‐ and post‐test CPP scores in all groups (Two‐way ANOVA test, F _(3,60)_ = 5.97, Sadik's multiple comparisons test: Low dose, *p* < 0.01; Medium, *p* < 0.0001; High, *p* < 0.001, Figure 1B). These results suggest that caffeine induces CPP, particularly at medium and high doses, highlighting its addictive properties.

**TABLE 1:** CPP scores in mice induced by different doses of caffeine.

	Pre‐test	Post‐test
Low dose (0.16 mg/kg)	−59.12	53.33
Medium dose (0.32 mg/kg)	−121.0	−1.312
High dose (0.64 mg/kg)	−61.17	88.19

### 3.2 Caffeine‐Induced 3D Behavioural Profiling and Motion Analysis

Behavioural profiling of caffeine‐treated mice (0.32 mg/kg) revealed notable differences compared to the saline control group. We selected the medium dose of 0.32 mg/kg as this dose produced a robust CPP response. Using advanced 3D motion analysis (Figure 1C), it was observed that caffeine‐treated mice exhibited more diverse and frequent behaviours, as visualised in the behavioural movement type and kinematic analysis (Figure 1D,F). Motion parameter analysis showed significant increases in tail swing velocity (Unpaired *t* test, *p* = 0.025), time spent in the central region (Unpaired *t* test, *p* = 0.028) and body length (Unpaired *t* test, *p* = 0.002) in the caffeine group. Specific actions such as increased instances of 10# sprinting (Unpaired *t* test, *p* = 0.02) and 36# forelimb rearing (Unpaired *t* test, *p* = 0.01) further differentiated caffeine‐treated mice from the saline group. The statistical results for each action are provided in Data S1. Dimensionality reduction and clustering of locomotor parameters demonstrated distinct distributions for caffeine and saline groups (Figure 1E), with minimal overlap, suggesting significant behavioural differences. These findings support the hypothesis that caffeine induces measurable changes in fine behavioural patterns, offering novel insights into its addictive potential.

### 3.3 Caffeine Activates the Medial Prefrontal Cortex

**FIGURE 2:**
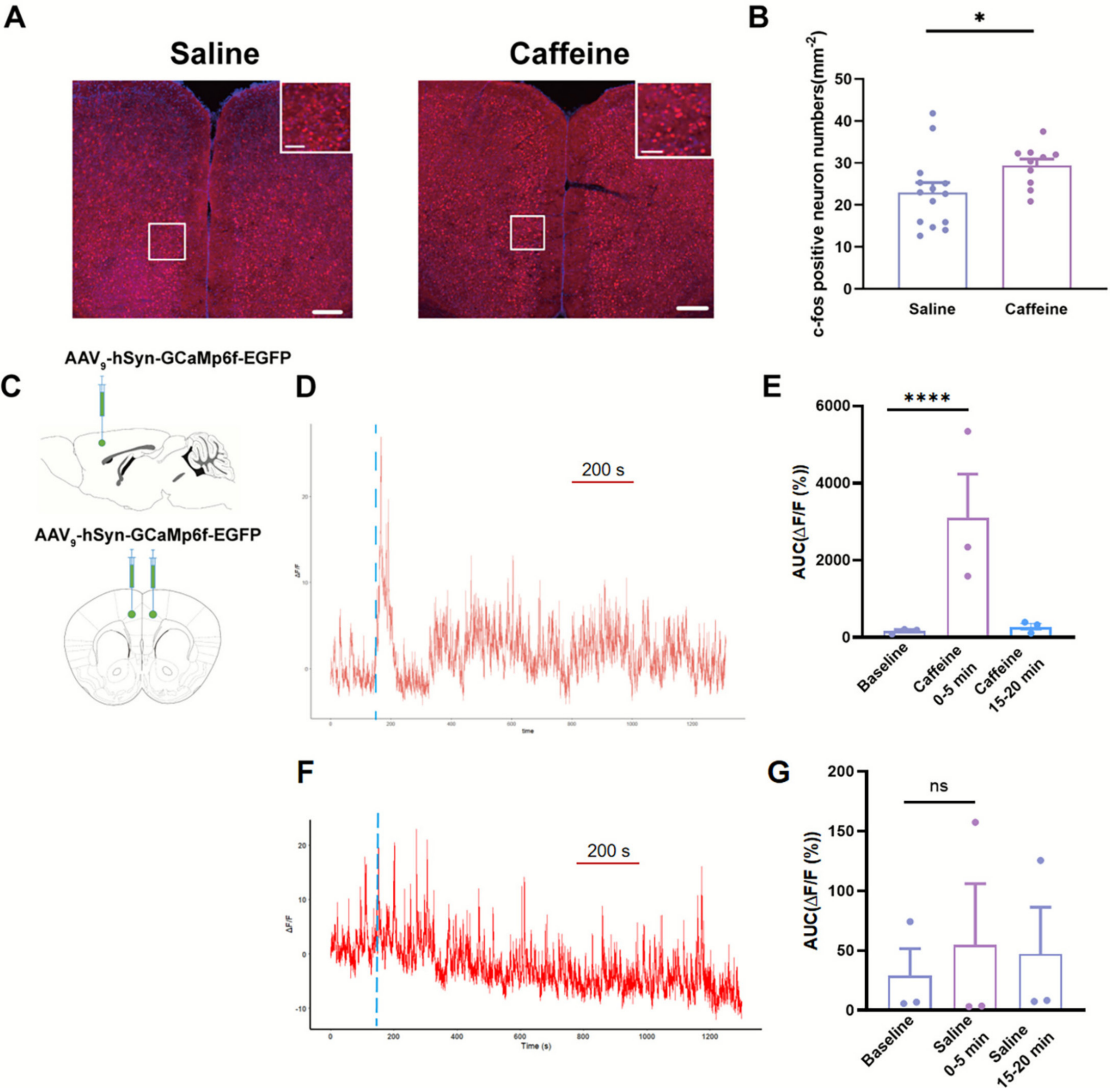
Caffeine‐induced neural activation of mPFC. (A) Representative images of c‐Fos^+^ neurons of the medial prefrontal cortex in the saline and caffeine groups. Top: the c‐Fos^+^ neurons of the medial prefrontal cortex in the saline group Bottom: c‐Fos^+^ neurons of the medial prefrontal cortex in the caffeine group. Scale bar: 100 μm. (B) The average percentage of c‐Fos^+^ in the saline and caffeine groups (saline group: *n* = 12 slice from 3 mice, caffeine group: *n* = 10 slice from 3 mice, Unpaired *t* test, *p* < 0.05). (C) Diagram of injection site in the medial prefrontal cortex. (D) Representative image of calcium signalling with caffeine effects. The blue line represents the timing of caffeine administration. (E) The average AUC of ΔF/F calcium signalling with caffeine effects (*n* = 3, Paired *t* test, *p* < 0.05). (F) Representative image of calcium signalling with saline effects. The blue line represents the timing of saline administration. (G) The average AUC of ΔF/F calcium signalling with saline effects (*n* = 3, Paired *t* test, *p* < 0.05). *p < 0.05, ****p < 0.0001 and Means ± SEMs.

To investigate the neural mechanisms underlying caffeine‐induced behaviour, immunohistochemical analysis revealed a significant increase in c‐fos expression in the medial prefrontal cortex (mPFC) of caffeine‐treated mice compared to saline controls, indicating enhanced neuronal activation in this region (Figure 2A,B). This elevation in c‐fos expression suggests that caffeine modulates neuronal activity within the mPFC, a brain region critically involved in executive functions [25], decision‐making [26] and reward processing [27].

Additionally, calcium signalling in the mPFC was monitored for 60 min following caffeine (0.32 mg/kg) administration to assess real‐time neuronal activity dynamics. During the first 5 min post‐injection, calcium signals exhibited a modest but measurable increase relative to baseline (Figure 2D,E), potentially reflecting heightened synaptic activity and neurotransmitter release driven by caffeine's stimulant effects. Interestingly, these calcium levels returned to baseline during the latter half of the recording period, indicating a transient nature of caffeine's excitatory influence on neuronal activity in the mPFC. In contrast, administration of saline had no effect on calcium signalling in the mPFC (Figure 2F,G). These findings provide a mechanistic link between caffeine's behavioural effects and its capacity to transiently enhance neuronal activity in key brain regions associated with arousal, attention and cognitive processing.

### 3.4 Transcriptomic Analysis of the mPFC

**FIGURE 3:**
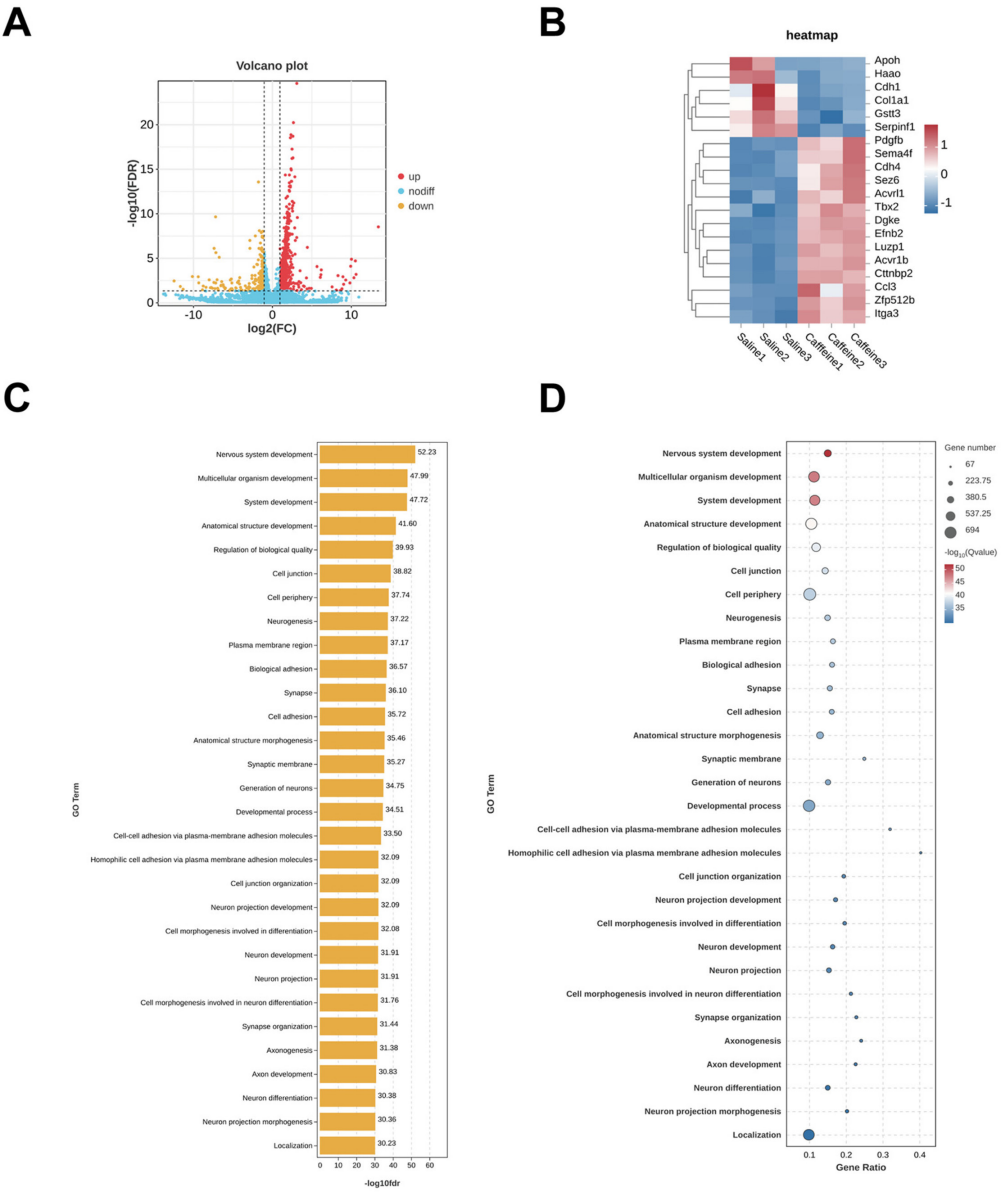
RNA‐Seq analysis of caffeine‐induced changes of mPFC. (A) The Volcano Plot of upregulated and downregulated genes in the saline‐treated and caffeine‐treated group. (B) The heatmap of upregulated and downregulated genes in the saline‐treated and caffeine‐treated group. (C) The grouped secondary bar chart of Gene Ontology (GO) enrichment analysis. (D) GO Enrichment Bubble Plot in the saline‐treated and caffeine‐treated group. All data from 5 mice.

Transcriptomic profiling of the mPFC in caffeine‐treated mice identified significant changes in gene expression. Volcano plots highlighted genes with upregulated and downregulated expression (Figure 3A) and heatmap of upregulated and downregulated genes in the saline‐treated and caffeine‐treated groups (Figure 3B). GO term analysis revealed significant enrichment in molecular functions such as transport activity, signalling processes and developmental pathways, reflecting caffeine's broad impact on cellular processes (Figure 3C,D). The observed changes suggest that caffeine influences not only immediate synaptic functions but also long‐term molecular pathways critical for neuronal adaptation and function.

**FIGURE 4:**
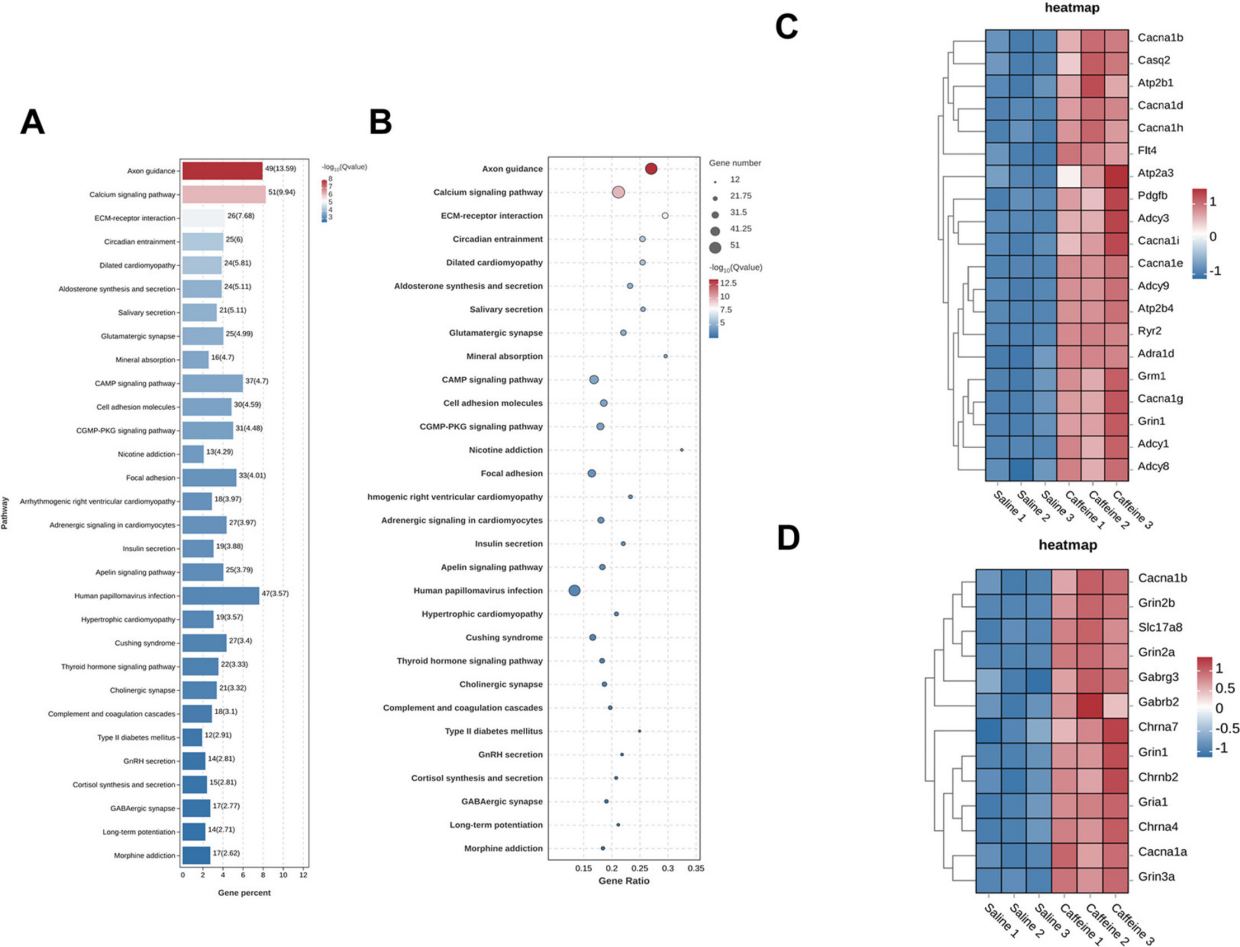
RNA‐Seq analysis of caffeine‐induced changes of mPFC. (A) KEGG enrichment bar chart between the saline‐treated and caffeine‐treated group. (B) KEGG Enrichment Bubble Plot in the saline‐treated and caffeine‐treated group. (C) Heatmap of target genes in the Calcium signalling pathway. (D) Heatmap of target genes in the Nicotine Addiction pathway. All data from 5 mice.

KEGG pathway analysis corroborated these findings, with pathways such as calcium signalling, synaptic transmission and addiction‐related pathways showing high significance. Among the most prominently altered pathways, notable enrichment in pathways related to calcium signalling and nicotine addiction (Figure 4A,B). Calcium signalling was linked to changes in synaptic plasticity and neurotransmitter regulation, providing molecular evidence for caffeine's effects on neuronal excitability. The heatmap of target genes in the Calcium signalling pathway is shown in Figure 4C. The heatmap of target genes in the Nicotine Addiction pathway is shown in Figure 4D. Notably, the overlap between pathways involved in calcium signalling and nicotine addiction highlights potential commonalities in how stimulant substances influence reward‐related and plasticity‐associated mechanisms. Together, these results suggest that caffeine modulates neural activity and gene expression in the mPFC, contributing to its behavioural and addictive effects, while providing valuable insights into its molecular mechanisms. The identified pathways establish a mechanistic foundation for subsequent targeted research aimed at delineating both the neurotherapeutic benefits and potential adverse consequences of caffeine on brain function.

## 4 Discussion

This study demonstrates that caffeine induces CPP, alters some kinematic parameters that are indicative of the psychostimulant effects of caffeine and modulates neural activity and gene expression in the mPFC, providing evidence for its addiction potential. The results confirm that caffeine, even at relatively low doses, can induce reward‐seeking behaviours, with medium and high doses producing the most pronounced effects. These findings align with the broader literature suggesting that caffeine shares behavioural and neurobiological properties with other addictive substances, such as psychostimulants in psychomotor stimulation and reinforcing stimuli [28, 29, 30, 31].

The observed CPP highlights caffeine's rewarding effects, particularly at medium (0.32 mg/kg) and high (0.64 mg/kg) doses, indicating a dose‐dependent response. Low doses also demonstrated significant differences pre‐ and post‐conditioning, though to a lesser extent. These dose‐dependent effects may reflect varying activation thresholds of dopamine‐dependent reward circuits. Caffeine's ability to induce CPP not only supports its reinforcing properties but also underscores the relevance of dose optimisation in studying substance addiction. The use of advanced 3D behavioural profiling provided a more nuanced understanding of caffeine's effects, revealing specific changes in locomotor activity, central zone exploration and body dynamics. These results suggest that caffeine alters both the macro‐level behaviours and micro‐level movement patterns in ways similar to other stimulants, contributing to its potential for habitual use.

The mPFC is critically involved in reward processing, decision‐making [26] and habit formation [32], while the hippocampus contributes to contextual memory [33] and emotional regulation [34]. The temporal dynamics of calcium signalling—initially heightened and later stabilising—may reflect transient neural adaptations during caffeine exposure. These adaptations could correspond to changes in synaptic plasticity and neurotransmitter release, critical processes in addiction development [35, 36].

To exclude the possibility that the observed calcium transients were caused by injection‐related artefacts, we included a saline control group in the revised experiments. The ΔF/F signals from saline‐injected mice remained stable (mean change < 5%), indicating that the calcium activity increases were specific to caffeine administration. Moreover, the temporal profile of the caffeine‐evoked calcium response—characterised by a rapid rise peaking at approximately 5 min and returning to baseline within 20 min—corresponds well with reported caffeine pharmacokinetics in mice [37]. These findings support that the recorded calcium dynamics reflect genuine neuronal responses to caffeine exposure.

Caffeine's modulation of calcium signalling is particularly significant, given the established role of calcium‐dependent pathways in synaptic transmission and plasticity [38, 39]. This finding bridges behavioural observations with cellular mechanisms, suggesting that caffeine's addictive properties may be partly mediated through alterations in calcium homeostasis and related intracellular signalling cascades.

Transcriptomic analysis further supports caffeine's complex impact on brain function, revealing upregulation of genes involved in calcium signalling, synaptic function and pathways linked to nicotine addiction. The overlap between caffeine and nicotine‐related pathways highlights shared molecular mechanisms that may underlie the addictive properties of stimulants. This finding is particularly relevant in the context of poly‐substance use, as individuals consuming caffeine may be more susceptible to nicotine or other substance addictions [40, 41, 42].

The significant enrichment of genes within GO terms related to developmental processes and transport activities suggests that caffeine may influence neurodevelopmental processes or neurotransmitter transport systems. This raises intriguing questions about the long‐term effects of caffeine exposure, particularly during critical developmental windows. Furthermore, the KEGG pathway analysis emphasizes the prominence of calcium signalling and related pathways, providing targets for future research into therapeutic interventions.

While this study offers valuable insights, several limitations must be addressed. First, the focus on a single brain region (mPFC) provides an incomplete picture of the neural circuits involved in caffeine addiction. Future studies should incorporate other regions, such as the striatum and ventral tegmental area, to elucidate the broader network dynamics. Additionally, while the study identifies significant transcriptomic changes, functional validation of these genes is necessary to confirm their roles in caffeine‐induced behaviours. Techniques like CRISPR‐based gene editing or chemogenetics could provide causal evidence for specific gene contributions [24].

Moreover, the behavioural paradigms used, while robust, may not fully capture the complexities of caffeine addiction in humans. Translating these findings to clinical settings requires consideration of human‐specific factors such as genetic predisposition, environmental influences and poly‐substance use patterns. Future research could also explore the interplay between caffeine and stress or other environmental factors, which are known to influence addiction vulnerability [43, 44, 45].

Understanding caffeine's addictive potential has broad implications for public health and neuroscience. As caffeine is widely consumed and often perceived as benign, its potential for addiction and neuroplastic changes warrants further scrutiny. These findings could inform guidelines for safe caffeine consumption and provide a basis for public health interventions targeting at‐risk populations.

Several limitations should be noted. First, the present work focused on short‐term caffeine exposure; long‐term administration and withdrawal effects remain to be evaluated. Second, only one caffeine dose was tested in the fibre photometry experiments, which may not capture dose‐dependent neural responses. Third, only male mice were used, and potential sex differences in caffeine sensitivity and reward remain unexplored. Finally, future work employing self‐administration or reinstatement paradigms would help elucidate caffeine's addictive potential under more naturalistic conditions.

In conclusion, this study provides a comprehensive examination of caffeine's behavioural, neural and molecular effects, shedding light on its addictive properties and underlying mechanisms. By integrating behavioural analyses with neural and transcriptomic data, this work offers a foundation for future research aimed at mitigating the risks associated with caffeine consumption while exploring its potential as a model for studying stimulant addiction.

## Author Contributions

Y.G., X.L. and F.F. conceived the study. Y.G. and X.L. designed the experiments. X.L. and X.L. carried out and analysed behaviour and biochemical experiments, and X.L., F.F. and Y.G. wrote the manuscript. P.C., H.S., Y.H. and Y.S. carried out and analysed stereotaxic surgery experiments. X.L. and F.F. analysed transcriptomic data. This study may provide a theoretical foundation for future investigations into shared and distinct mechanisms among different addictive substances. Authors read and approved the final manuscript.

## Funding

The authors have nothing to report.

## Conflicts of Interest

The authors declare no conflicts of interest.

## Supporting information


**Data S1:** Supporting information.

## Data Availability

The datasets used and/or analysed during the current study are available from the corresponding author on request.
